# Pharmacokinetic/Pharmacodynamic Evaluation of a New Purine-2,6-Dione Derivative in Rodents with Experimental Autoimmune Diseases

**DOI:** 10.3390/pharmaceutics14051090

**Published:** 2022-05-19

**Authors:** Artur Świerczek, Krzysztof Pociecha, Hanna Plutecka, Marietta Ślusarczyk, Grażyna Chłoń-Rzepa, Elżbieta Wyska

**Affiliations:** 1 Department of Pharmacokinetics and Physical Pharmacy, Faculty of Pharmacy, Jagiellonian University Medical College, 9 Medyczna Street, 30-688 Krakow, Poland; k.pociecha@uj.edu.pl; 2 Department of Internal Medicine, Faculty of Medicine, Jagiellonian University Medical College, 8 Skawińska Street, 31-066 Krakow, Poland; hanka.plutecka@uj.edu.pl; 3 Department of Medicinal Chemistry, Faculty of Pharmacy, Jagiellonian University Medical College, 9 Medyczna Street, 30-688 Krakow, Poland; marietta.slusarczyk@doctoral.uj.edu.pl (M.Ś.); grazyna.chlon-rzepa@uj.edu.pl (G.C.-R.)

**Keywords:** disease progression modeling, phosphodiesterase inhibitors, rheumatoid arthritis, multiple sclerosis, autoimmune hepatitis

## Abstract

Current treatment strategies of autoimmune diseases (ADs) display a limited efficacy and cause numerous adverse effects. Phosphodiesterase (PDE)4 and PDE7 inhibitors have been studied recently as a potential treatment of a variety of ADs. In this study, a PK/PD disease progression modeling approach was employed to evaluate effects of a new theophylline derivative, compound **34**, being a strong PDE4 and PDE7 inhibitor. Activity of the studied compound against PDE1 and PDE3 in vitro was investigated. Animal models of multiple sclerosis (MS), rheumatoid arthritis (RA), and autoimmune hepatitis were utilized to assess the efficacy of this compound, and its pharmacokinetics was investigated in mice and rats. A new PK/PD disease progression model of compound **34** was developed that satisfactorily predicted the clinical score-time courses in mice with experimental encephalomyelitis that is an animal model of MS. Compound **34** displayed a high efficacy in all three animal models of ADs. Simultaneous inhibition of PDE types located in immune cells may constitute an alternative treatment strategy of ADs. The PK/PD encephalomyelitis and arthritis progression models presented in this study may be used in future preclinical research, and, upon modifications, may enable translation of the results of preclinical investigations into the clinical settings.

## 1. Introduction

Cyclic adenosine 3′, 5′-monophosphate (cAMP), and cyclic guanosine 3′, 5′-monophosphate are cyclic nucleotides (cNMPs) that play a key role as second messengers in many cellular processes like metabolism, gene expression, and calcium homeostasis. Their intracellular concentration is regulated by the activity of three enzymes: adenylyl cyclase and guanylyl cyclase, which increase the levels of cNMP, and phosphodiesterase (PDE), which catalyzes the hydrolysis of cNMP. Adenylyl cyclase is activated by G protein-coupled receptors that bind to the extracellular ligands [1,2]. Up to date, there are 11 families of PDEs described in literature that differ in sequence homology and substrate affinity. One of the main downstream effectors of cAMP is a protein kinase A, which modulates the activity of nuclear factor kappa-light-chain-enhancer of activated B cells. This transcription factor has been identified as the key player in the immune response, cytokine production, and gene transcription [3,4]. Having in mind the crucial role of cNMP in controlling of biological functions, PDEs are important drug targets in a variety of disorders. Recent studies suggest that targeting PDE4 and PDE7 isoenzymes, which are predominantly present in immune cells, is an attractive approach for alleviating autoimmune diseases (ADs) [5,6].

Multiple sclerosis (MS) is a severe demyelinating disease of autoimmune background. It is estimated that 2.8 million people live currently with MS worldwide. Development of MS leads to disability at a productive stage of life and impacts both diagnosed patients and their families [7]. Experimental autoimmune encephalomyelitis (EAE) is a well-established animal model of human MS that helps in elucidating the pathomechanism of this disease and developing novel disease-modifying drugs [8]. EAE is induced in animals by administration of central nervous system (CNS) proteins, such as myelin oligodendrocyte glycoprotein (MOG), coupled to adjuvant. Inoculation triggers the immune response of autoreactive T-cells, which migrate through the blood–brain barrier and interact with antigen presenting cells like micoglia or astrocytes. Auto-responsive T-cells undergo a process called maturation and promote the development of CNS inflammation and damage [9,10]. It has been proved that elevated levels of intracellular cAMP achieved through PDE4 inhibition can lead to attenuation of EAE clinical signs, since PDE4 is predominantly present in immune cells [8,11]. Recent studies show that PDE7 inhibitors are also a viable target for the therapeutic approach for EAE, especially considering their lack of emetic properties that are a major drawback of potent, PDE4-selective inhibitors [12,13,14,15]. It is also believed that other PDEs, such as PDE1 or PDE3, that are present in immune cells may support beneficial effects of PDE4 and PDE7 in some inflammatory diseases [16].

Rheumatoid arthritis (RA) is an AD that affects synovial joints and other body parts, i.e., skin, lungs, and heart. RA is a major cause for chronic pain and disability and could lead to a reduced lifespan. It is believed that pro-inflammatory cytokines like tumor necrosis factor α (TNFα) and interleukin 1 (IL-1) are key factors in the development of joint inflammation and clinical symptoms [17]. Treatment with a chimeric anti-TNFα monoclonal antibody (mAB) resulted in amelioration of disease severity in clinical trials. Nonetheless, it is believed that prolonged therapy with mAB may reduce its effectiveness, therefore, alternative anti-TNFα approaches are being developed. Inhibiting cAMP hydrolysis is an example of those attempts. In animal models of RA, elevating the intracellular cAMP by administration of PDE4 inhibitors resulted in suppression of TNFα expression and incidence of disease symptoms [18].

An intravenous (IV) injection of concanavalin A (ConA) in mice is a widely used animal model for autoimmune hepatitis (AIH). It was demonstrated that ConA-induced hepatitis is moderated by helper T-cells, Kupffer cells, and natural killer T cells, which induce apoptosis of hepatocytes by Fas-FasL interaction and IL-4 release [19]. Recent studies have shown that PDE7A is widely expressed in natural killer T cells, and its inhibition by a specific inhibitor leads to a reduction in IL-4 production and liver injury. In addition, a decrease in TNFα concentration was also observed [20,21].

Mechanistic pharmacokinetic/pharmacodynamic (PK/PD) and disease progression modeling are a rapidly expanding field that aids in the development of new drugs and treatment strategies. Mathematical models describing the relationship between drug exposure and its effects, combined with models of disease progression, are helpful not only in the quantitative assessment of potential drug candidates and translation of the results of preclinical investigations into the clinical settings but also in better understanding determinants of drug’s action and pathological mechanisms leading to disease development [22,23]. These issues are especially important in studies involving ADs, whose pathophysiology is still not completely elucidated, and new effective and safe therapies of these diseases are constantly being sought out. 

**Figure 1:**
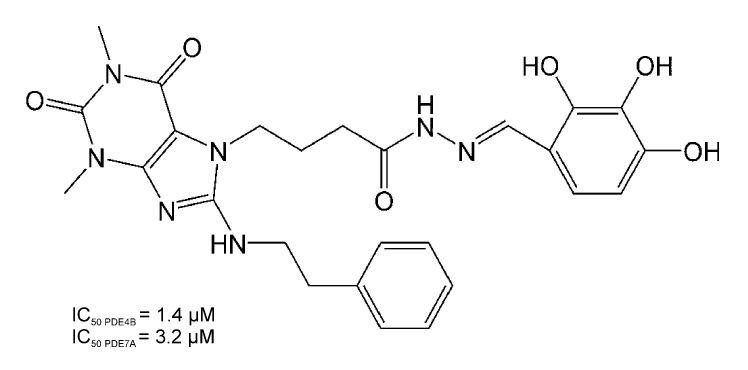
Chemical structure of compound **34**; 4-(1,3-dimethyl-2,6-dioxo-8-(phenethylamino)-2,3,6,7-tetrahydro-1*H*-purin-7-yl)-*N*′-(2,3,4-trihydroxybenzylidene)butanehydrazide.

The aim of this study was to assess the effects of a new purine-2,6-dione derivative, compound **34** (Figure 1), being a strong inhibitor of PDE4 and PDE7 in animal models of ADs, namely MOG_35–55_-induced EAE in mice, which is a model of MS [10], collagen-induced arthritis (CIA) in rats, which is a model of RA [24], and ConA-induced hepatitis in mice that, in many aspects, resembles human AIH [20].

To this end, a PK/PD disease progression modeling approach was utilized that enabled us to mathematically describe the relationship between compound **34** pharmacokinetics, its pharmacological effects, and the influence on disease progression. The proposed PK/PD arthritis and EAE progression models of compound **34** allowed for quantitative assessment of compound **34** potency in vivo. In addition, the ability of the studied PDE4/7 inhibitor to interact with other representatives of PDE family that are present in immune cells and may contribute to the observed effects was evaluated in vitro.

## 2. Materials and Methods

### 2.1. Chemicals and Reagents

Compound **34** was designed and synthetized in the Department of Medicinal Chemistry, Faculty of Pharmacy, Jagiellonian University Medical College, Kraków, Poland [25]. ConA was purchased from Santa Cruz Biotechnology (Dallas, TX, USA). PEG400 and DMSO were obtained from Sigma-Aldrich (Steinheim, Germany). All other reagents, chemicals, and solvents were of HPLC or analytical grade and were purchased from Merck (Darmstadt, Germany).

### 2.2. Animals

Female Lewis rats (7–8 weeks old) weighing 150–180 g and female C57BL/6 mice (9–11 weeks old) weighing 18–26 g purchased from Mossakowski Medical Research Center Polish Academy of Sciences, Warsaw, Poland, and female BALB/c mice (8–12 weeks old) weighing 18–22 g obtained from the Animal Facility of the Department of Clinical Immunology and Transplantology, Jagiellonian University Medical College, Kraków, Poland, were used in this study. The animals were housed in the Laboratory Animal Facility of the Faculty of Pharmacy, Jagiellonian University Medical College, Kraków, Poland, under constant temperature of 21 °C and humidity of 50% (±10%). They were exposed to 12:12 light-dark cycle and maintained on a standard pellet diet and tap water given ad libitum. The investigated compound was administered intraperitoneally (IP) after dissolution in a mixture of DMSO:PEG400:sterile water (10:40:50) in mice and as a suspension in 10% methylcellulose in rats. Control animals received the equivalent volume of vehicles by the same route of administration.

### 2.3. Experimental Procedures

#### 2.3.1. PDE Assay

The inhibitory activity of compound **34** against hrPDE1B and hrPDE3A (SignalChem, Richmond, Canada) was assessed using the PDE-Glo™ catalytic activity assay (Promega Corporation, Madison, WI, USA). It was performed on 384-well plates according to manufacturer’s instructions with slight modifications. Solutions of both PDEs were prepared by diluting their stock solutions in the PDE-Glo™ Reaction Buffer and aliquots of 6.5 μL were added to the wells. A stock solution of the tested compound was prepared in DMSO at a concentration of 10 mM and serially diluted with the same solvent. The obtained solutions were further diluted with PDE-Glo™ Reaction Buffer (1:5, *v*/*v*) and aliquots of 1 μL were transferred to the respective wells. Then, the mixture was incubated for 10 min at 30 °C on a heated plate shaker (Grant Instruments, Cambridge, United Kingdom). The reaction was started by the addition of 2.5 μL of a 0.2 μM cAMP solution to each well and the plate was incubated for 10 min at 30 °C on a heated plate shaker. Moreover, for each series, respective control reactions were performed: no-substrate, no-enzyme negative control reactions; no-enzyme positive control reactions containing substrate; and no-inhibitor positive control reactions containing both the substrate and PDE. After incubation, the reaction was stopped by adding 2.5 μL of the PDE-Glo™ Termination Buffer. A volume of 2.5 μL of the PDE-Glo™ Detection Buffer was transferred to each well and the plate was incubated for 20 min at room temperature. Finally, an aliquot of 10 μL of the PDE-Glo Kinase^®^ Reagent was added to the wells. The plate was incubated for 10 min at room temperature and luminescence was measured with a luminometer (BMG Labtech, Ortenberg, Germany). For each inhibitor concentration, four independent measurements were performed. The resulting data were expressed as percent of an uninhibited control and plotted against the inhibitor concentration. The IC_50_ values were estimated by nonlinear regression using ADAPT5 software (BMSR, Los Angeles, CA, USA).

#### 2.3.2. Pharmacokinetic Study

Female Lewis rats and female C57BL/6 mice were administered IP with compound **34** dissolved in vehicle at a dose of 20 and 50 mg·kg^−1^, respectively. Rats and mice were sacrificed at different time points (*n* = 3 per time point) following compound administration by decapitation under deep anesthesia. Thereafter, blood samples were harvested and allowed to clot at room temperature for 20 min and serum was separated by centrifugation at 3000 rpm for 10 min (EBA 12 R, Hettich, Germany). The obtained serum samples were stored at −80 °C until analysis.

#### 2.3.3. Collagen-Induced Arthritis

Female Lewis rats were subcutaneously injected at the base of the tail with the emulsion prepared according to the protocol supplied by Chondrex, Inc. (Redmond, WA, USA) at days 0 and 7 of the experiment. To obtain the emulsion, porcine collagen type II (Chondrex, Inc.) (2 mg/mL) in 0.05 M acetic acid was emulsified with incomplete Freund’s adjuvant (Sigma-Aldrich, St. Louis, MO, USA) using a homogenizer with a small blade (LabGen 125, Cole-Parmer, Vernon Hills, IL, USA). On day 20, the animals that developed symptoms of arthritis, i.e., paw swelling above 150% of day 0, were randomly divided into two groups: the control group receiving vehicle alone (*n* = 6) and the study group treated with the investigated compound at a dose of 20 mg·kg^−1^, IP once daily for 21 days (*n* = 6). Relative paw edema of arthritic rats was measured using a digital caliper according to the procedure described by Earp et al. [26] up to 40 days after the first immunization and then the animals were sacrificed. In addition, the body weight of animals was monitored. After randomization on day 20, using the Mann–Whitney U test, it was verified that there is no significant difference in paw size between the groups.

#### 2.3.4. Experimental Autoimmune Encephalomyelitis

Female C57BL/6 mice were immunized by subcutaneous injection of MOG_35–55_ peptide emulsified in complete Freund’s adjuvant and IP administration of pertussis toxin solution in PBS (Hooke Laboratories, Inc., Lawrence, MA, USA). Mice developed EAE 8–14 days after immunization and on day 14 they were randomly divided into two groups. The study group (*n* = 6) was administered with compound **34** solution at an IP dose of 50 mg·kg^−1^ once daily for 15 days, whereas the control group (*n* = 6) received vehicle alone. To follow the progress of the disease, clinical score (*CS*) (scale 0–5), including “in-between” scores (i.e., 0.5, 1.5, 2.5, 3.5, and 4.5), and body weight of mice were evaluated according to the protocol provided by Hooke Laboratories, Inc. [27]. Using the Mann–Whitney U test, it was verified that there is no significant difference in *CS* between the groups on the day of randomization. Finally, the animals were sacrificed on the 28th day post-immunization.

#### 2.3.5. ConA-Induced Hepatitis

ConA was dissolved in saline for injection at a concentration of 2 mg·mL^−1^. The mice were given IP doses of compound **34** or the equivalent volume of vehicle (control group) and, 30 min later, animals were given an IV injection of ConA (20 mg·kg^−1^) via tail vein. An additional group of mice was given vehicle alone IP followed by an IV injection of saline. The mice were sacrificed by exsanguination at 2, 8, and 24 h post-ConA dosing. Blood samples were collected and handled as described above.

### 2.4. Analytical Methods 

Concentrations of compound **34** in rat and murine serum were quantified by an HPLC/UV method. To isolate the investigated compound, 100 μL of serum containing compound **34** or the same volume of blank serum spiked with a standard solution of compound **34**, 10 μL of 4-(8-((furan-2-ylmethyl)amino)-1,3-dimethyl-2,6-dioxo-2,3,6,7-tetrahydro-1*H*-purin-7-yl)-*N*’-(2-hydroxybenzylidene)butanehydrazide methanol solution was used as an internal standard (IS) at a concentration of 80 μg·mL^−1^, and 50 μL of 0.2 M NaOH solution was added. Subsequently, 1 mL of ethyl acetate was added to each sample and extraction was performed for 20 min on a VXR Vibrax shaker (IKA, Germany). Then, the samples were centrifuged (10,000× *g*, 5 min) on EBA 12 R centrifuge (Hettich, Germany) and the organic layers were collected and transferred to clean tubes. Subsequently, the organic layers were evaporated under a mild stream of nitrogen at 37 °C and the residues were dissolved in 100 μL of mobile phase and subjected to an HPLC analysis. The HPLC system (LaChrom Elite, Merck-Hitachi, Germany) consisted of L-2420 UV-VIS detector, an L-2200 autosampler, and an L-2130 pump. EZChrome Elite v. 3.2 (Merck-Hitachi, Germany) software was used for data acquisition and integration. The analysis was performed at ambient temperature on a LiChrospher 100 RP-18 column (250 × 4 mm) and a LiChroCART (4 × 4 mm) pre-column (Merck, Germany). The mobile phase was composed of acetonitrile mixed with 20 mM KH_2_PO_4_ water solution (pH = 4.5) at the ratio of 40:60 (*v*/*v*). A flow rate was 1 mL·min^−1^ and analytical wavelength was set at 302 nm. The retention times of compound **34** and IS were approximately 6.3 min and 10.6 min. The method was validated according to the FDA Bioanalytical Method Validation Guidance for Industry (2018). The calibration curves constructed by plotting the peak area of the studied compound to the peak area of the IS vs. compound concentrations were linear in the tested concentration range, i.e., from 0.05–10 mg·L^−1^ (r > 0.998). The limit of quantification was 0.05 mg·L^−1^. No interfering peaks were observed in the chromatograms, indicating the high selectivity of the developed method. The assay presented acceptable intra- and inter-assay precision and accuracy (i.e., they were within 15% of the CV and 15% of the nominal concentration, respectively) for all quality control concentrations (0.1, 1, and 5 mg·L^−1^). The mean extraction efficiencies of the analyzed compound and the IS were above 80%.

Serum aspartate transaminase (AST) and alanine transaminase (ALT) activities were analyzed using the chemistry analyzer BS-800 (Shenzhen Mindray Bio-medical Electronics Co., Ltd., Shenzhen, China). Cytokine levels in mouse serum (TNFα, IL-4, IL-6, and interferon (IFN)γ) were quantified by a Magnetic Luminex^®^ Assay (R&D Systems, Inc, Minneapolis, MN, USA) using a Magpix platform (Luminex Corp., Austin, TX, USA).

### 2.5. Data Analysis

#### 2.5.1. Pharmacokinetics

Pharmacokinetics of compound **34** were investigated in rats and mice following single IP administration. One- and two-compartment models with first-order absorption and linear elimination were tested, but only the one-compartment model appropriately described the concentration-time courses of compound **34** in both rat and murine serum. In the case of the two-compartment model, the algorithm did not achieve convergence. The equations of the model are as follows:

(1) $$\frac{dA}{dt}=-k_{a}\times A;A\left(0\right)=D\times F,$$

(2) $$\begin{array}{c} V_{d}\times \frac{dC_{p}}{dt}=k_{a}\times A-CL_{T}\times C_{p};\;\;C_{p}\left(0\right)=0 \end{array}$$

where *A* is the amount of the drug at the absorption site, *k_a_* is the first-order rate constant of drug absorption, *D* is the dose, *F* is the bioavailability, *V_d_* is the volume of distribution, *CL_T_* is the total clearance, and *C_p_* is the serum concentration of compound **34**.

#### 2.5.2. PK/PD CIA Progression Model of Compound **34**

**Figure 2:**
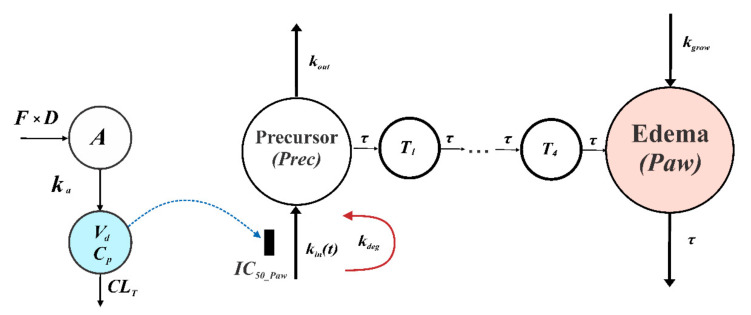
Schematic presentation of the proposed PK/PD CIA progression model of compound **34** in rats.

A previously developed PK/PD model of arthritis progression in rats was utilized in this study (Figure 2). The model was described in detail in our previous publication [28]. Originally, this model was used to assess and compare the effects of a PDE4-selective inhibitor rolipram and two non-selective PDE inhibitors, namely GRMS-55 and lisofylline in CIA in female Lewis rats. 

Briefly, the model assumes that PDE inhibitors act by suppressing the production of a precursor of arthritis progression. This is in line with the actual mechanism of anti-inflammatory action of PDE inhibitors that exert their effects by inhibiting the transcription of pro-inflammatory mediators, which participate in the arthritis development. The amount of the precursor of arthritis progression (*Prec*) is transmitted by the series of transit compartments (*T*_1_–*T*_4_), and the signal from the 4th transit compartment accounts for the production of paw edema that is represented by the relative value (*Paw*). In addition, natural paw growth is represented by the zero-order rate constant *k_grow_*. In the present study, the same strain and sex of rats was used in both PK and PD experiments as in our previous investigation. Therefore, it was feasible to compare the potency of compound **34** and other PDE inhibitors that were evaluated in our previous study [28]. The model structure is described by the following set of differential equations:

(3) $$\frac{dPrec}{dt}=\left\{\begin{array}{c} \begin{array}{l} 0,\;\;\;\;\;\;\;\;\;\;\;\;\;\;\;\;\;\;\;\;\;\;\;\;\;\;\;\;\;\;\;\;\;\;\;\;\;\;\;\;\;\;\;\;\;\;\;\;\;\;\;\;\;\;\;\;\;\;\;\;\;\;\;\;\;\;\;\;\;\;\;\;\;\;\;\;\;\;\;\;\;\;\;\;t<t_{onset},\\ k_{in}\left(t\right)\cdot \left(1-\frac{C_{p}}{IC_{50\_Paw}+C_{p}}\right)-k_{out}\cdot Prec,\;\;t\geq t_{onset}, \end{array} \end{array}\right.Prec\left(0\right)=1$$

(4) $$\frac{dk_{in}}{dt}=-k_{deg}\cdot k_{in}\left(t\right);k_{in}\left(0\right)=k_{in}^{0}$$

where *k_in_*(*t*) is the variable representing the production of *Prec*, the initial condition of *k_in_*, namely $k_{in}^{0}$ was estimated for each group of rats, *k_out_* is the first-order rate constant of *Prec* degradation, the first immunization was performed at time (*t*) 0, *t_onset_* is the time of disease onset, *IC*_50_*Paw*_ is the concentration of compound **34** in serum producing 50% of inhibition of *Prec* production. The model assumes the maximum inhibition of *Prec* production of 100% by the studied compound. It has to be mentioned that the PK/PD model uses the total concentration of the compound in serum and does not take into consideration a possible impact of protein binding in serum or tissues. The first-order rate constant, *k_deg_*, accounts for the *k_in_* variable decay. The *Prec* is transmitted by the series of transit steps as follows:

(5) $$\frac{dT_{1}}{dt}=\left(Prec-T_{1}\right)\cdot \frac{1}{\tau };T_{1}\left(0\right)=1$$

where *T*_1_ is the first precursor in this series, *τ* is the transit time of the precursor between subsequent compartments, and, in addition, 1/*τ* serves as the first-order rate constant of paw edema reduction.

(6) $$\frac{dT_{n+1}}{dt}=\left(T_{n}-T_{n+1}\right)\cdot \frac{1}{\tau };\;T_{n+1}\left(0\right)=1,\;n=1\sim 3$$

*T_n_* is the *n*th precursor in the series of transit steps.

(7) $$\frac{dPaw}{dt}=\left(T_{4}-Paw\right)\cdot \frac{1}{\tau }+k_{grow};Paw\left(0\right)=1$$

Finally, *Paw* is the relative paw edema and *k_grow_* is the zero-order rate constant of the natural paw growth.

#### 2.5.3. PK/PD EAE Progression Model of Compound **34**

**Figure 3:**
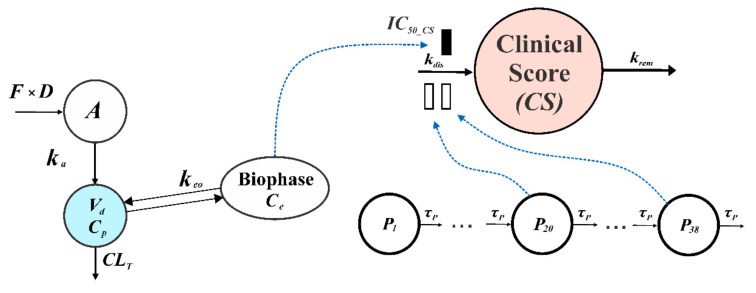
Schematic presentation of the proposed PK/PD MOG_35–55_-induced EAE progression model of compound **34** in mice.

To quantitatively assess the results obtained using the mouse model of MS, a *CS* was utilized as a marker of pharmacological response. A general schematic representation of the PK/PD EAE progression model of compound **34** is presented in Figure 3.

As can be seen from this figure, the model is composed of a one-compartment pharmacokinetic model describing serum concentrations of compound **34** that is combined with a biophase distribution model, which is linked to an indirect response model type I [29]. The model assumes that some time is required for the compound to distribute from the serum to the site of action before it interacts with a biological target and exerts the pharmacological effect. The gradual progression of EAE in mice was handled using a series of transit steps. The disease is initiated in the first transit compartment at time 0, which is the time of the immunization of animals. The value of the initial condition of the equation for first transducer (Equation (10)) was set to 1 for simplicity. The signal from the first transit compartment (*P*_1_) is transmitted through the series of transit compartments (*P*_2_–*P*_38_). The progression of disease, which was measured by *CS*, is stimulated by the sum of the signals from 20th and 38th transit compartments that allows us to capture the onset of the disease, the temporary remission, and the relapse of the disease that is a characteristic feature of this animal model of MS [30]. The total number of transit compartments, as well as the selection of the sum of signals from 20th and 38th transit compartments as a driving force of *CS* production, were determined by a trial-and-error method during the model development. Different combinations of numbers of transit compartments were tested and the optimal combination was selected based on goodness-of-fit criteria. The model equations and the initial conditions are as follows:

(8) $$C_{e}=(C_{p}-C_{e})\times k_{eo};\;C_{e}\left(0\right)=0$$

where *C_e_* is the concentration of the drug at the effect site, *k_eo_* is the first-order rate constant of equilibration of the drug between the serum and the site of action.

(9) $$\frac{dCS}{dt}=k_{dis}\cdot \left(P_{20}+P_{38}\right)\cdot \left(1-\frac{C_{e}}{IC_{50\_CS}+C_{e}}\right)-k_{rem}\cdot CS;\;CS\left(0\right)=0$$

*k_dis_* is the zero-order rate constant of *CS* production and *k_rem_* is the first-order rate constant of *CS* dissipation.

(10) $$\frac{dP_{1}}{dt}=-P_{1}\cdot \frac{1}{\tau_{P}};P_{1}\left(0\right)=1$$

*P_n_* is the *n*th precursor of disease progression and *τ_P_* is the transit time of the precursors between transit compartments. In addition, 1/*τ_P_* serves as the first-order degradation rate constant of the last precursor (*P*_38_).

(11) $$\frac{dP_{n+1}}{dt}=\left(P_{n}-P_{n+1}\right)\cdot \frac{1}{\tau_{P}};P_{n+1}\left(0\right)=0,n=1\sim 37$$

#### 2.5.4. Computation

ADAPT5 program (BMSR, University of South California, Los Angeles, CA, USA) was used to fit the PK and the PK/PD arthritis progression models to naïve-pooled data and mean *CS* values were utilized to fit the PK/PD EAE progression model. The model code for PK/PD EAE progression model is provided in the Supplementary Materials. The models were assessed based on the goodness-of-fit criteria, including visual inspection of the fitted profiles, analysis of residuals and observed vs. predicted values plots, Akaike Information Criterion, and coefficients of variation (CVs)% of parameter estimates. The variance model was set as:

(12) $$V_{i}={\left(\sigma_{1}+\sigma_{2}\cdot Y_{i}\right)}^{2}$$

where *V_i_* is the variance of the *i*th data point, *Y_i_* represents the *i*th model prediction, and *σ*_1_ and *σ*_2_ are the parameters of variance model. Initially, PK parameters were estimated and then they were fixed to run the PK/PD disease progression models. Plots and illustrations were prepared using OriginPro, Version 2022 (OriginLab Corporation, Northampton, MA, USA) and CorelDRAW^®^ Graphics Suite 2021 (Corel Corporation, Ottawa, ON, Canada).

#### 2.5.5. Statistical Analysis

The concentrations of inflammatory mediators, the body weight, and the activities of transaminases in serum were compared using a Student’s *t*-test. The normality of data distribution was assessed using a Shapiro–Wilk test. The statistical comparisons were performed in Statistica 13 (TIBCO Software Inc., Palo Alto, CA, USA). The significance level *p* was set at 0.05.

## 3. Results

### 3.1. PDE Assay

Inhibitory activities of compound **34** against human recombinant PDE1B and PDE3A were examined in this study. The *IC*_50_ values of the studied compound on PDE4B and PDE7A and reference compounds against PDE1B, PDE3A, PDE4B, and PDE7A were assessed in our previous investigations [25,28] and are summarized in Table 1.

All measurements presented in this table were performed by our research group within a one series of experiments under the same experimental conditions, thus, it is justified to compare the *IC*_50_ values with each other. As it can be seen in Table 1, compound **34** displayed a strong inhibitory activity against both PDE1B and PDE3A that was around 36 times higher than that of vinpocetine, a reference PDE1-selective inhibitor, and about 4 times lower compared to a potent PDE3-selective inhibitor milrinone. Moreover, compound **34** was 12 and 17 times more potent than its analog GRMS-55 in inhibiting PDE1B and PDE3A, respectively. It needs to be stressed that, according to the results of our previous studies, compound **34** has a similar inhibitory activity against PDE4 and PDE7 as the reference compounds, selective inhibitors of these enzymes, namely rolipram and BRL—50481.

### 3.2. Pharmacokinetics

**Figure 4:**
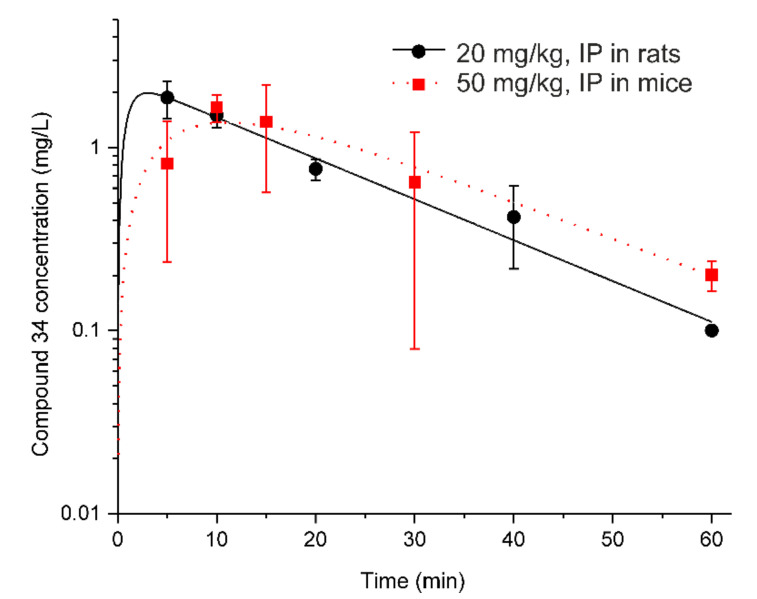
PK model fittings to compound **34** serum concentration vs. time profiles. Symbols represent the mean concentrations (±SD) of compound **34** in serum after its IP administration at a single dose of 20 mg·kg^−1^ in rats (*n* = 3 per time point) and at a single IP dose of 50 mg·kg^−1^ in mice (*n* = 3 mice per time point). Curves depict model fittings.

Figure 4 shows the results of the fitting of the PK model to the concentration-time data of compound **34** in rat and murine serum. From this figure, a one-compartment model with the first-order absorption and linear elimination satisfactorily described the experimental data. 

Compound **34** exhibited a rapid absorption from the intraperitoneal cavity with the absorption half-life of 1.2 min in rats and 4.5 min in mice. As it can be seen in Table 2, due to the fast absorption of the compound in rats and a lack of measured concentrations of the compound before 5 min post-injection, it was not possible to precisely estimate the *k_a_* of the drug in this species (CV% value for *k_a_* > 100%). The faster absorption in rats compared to mice may be due to different vehicles being used for drug delivery in both experiments. 

### 3.3. PK/PD Arthritis Progression Model

**Figure 5:**
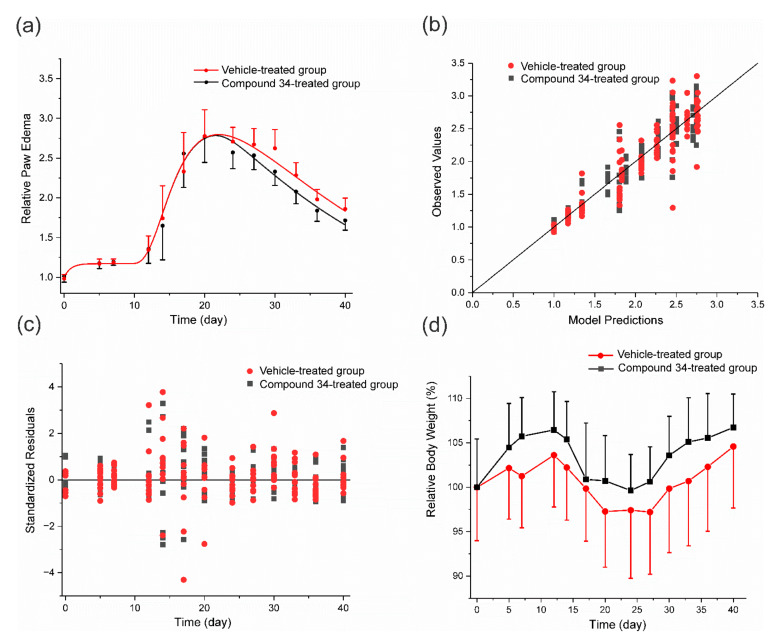
The fitting of the PK/PD arthritis progression model to the data and relative body weights of animals from both study groups; (**a**) time courses of the mean (±SD) relative paw edema in CIA rats (*n* = 6) after compound **34** treatment at an IP dose of 20 mg·kg^−1^ once daily for 21 days starting from the 20th day post-immunization, curves depict model fittings, and symbols are measured values; (**b**,**c**) are diagnostic plots of the PK/PD arthritis progression model; (**d**) time courses of the mean (±SD) relative body weight in arthritic rats from experimental and control groups (*n* = 6).

The results of the PK/PD arthritis progression model fitting to the data are shown in Figure 5. As can be seen from this figure, the model captured the experimental data well. Analysis of residuals and observed versus predicted values confirms that the model reasonably described the time courses of the relative paw edema in both experimental and control groups. A sharp rise in paw edema was observed on the 12th day post-immunization, it reached the peak value around the 20th day, and then gradually decreased due to natural remission of the disease. 

Relative body weight was not significantly different between the experimental and control groups during the experiment. There was an initial increase in body weight observed in both groups that may be attributed to the natural growth of rats and a subsequent drop due to disease progression. After the 24th day of the experiment, due to the natural remission of the disease, a gradual increase in body weight was observed. Estimated values of PD and disease progression parameters are presented in Table 3. 

The time of disease onset was estimated to occur on the 9th day post-immunization, but the paw swelling was observed after the 10th day. This delay is due to the signal transduction that links precursor of arthritis with the production of edema. This feature of the model allows to capture the time that is required for pro-inflammatory cytokine production (transcription and translation), release, and activation of signaling pathways leading to the swelling of joints. The estimated by the model *IC*_50_*Paw*_ value of compound **34** is relatively low and equals 0.001 mg·L^−1^, indicating a high in vivo potency of the compound; however, it has to be noted that it was estimated with a high uncertainty, i.e., CV% is above 300%. The high CV% may arise from the fact that only one relatively low dose of compound **34** was used in this study.

### 3.4. PK/PD EAE Progression Model

**Figure 6:**
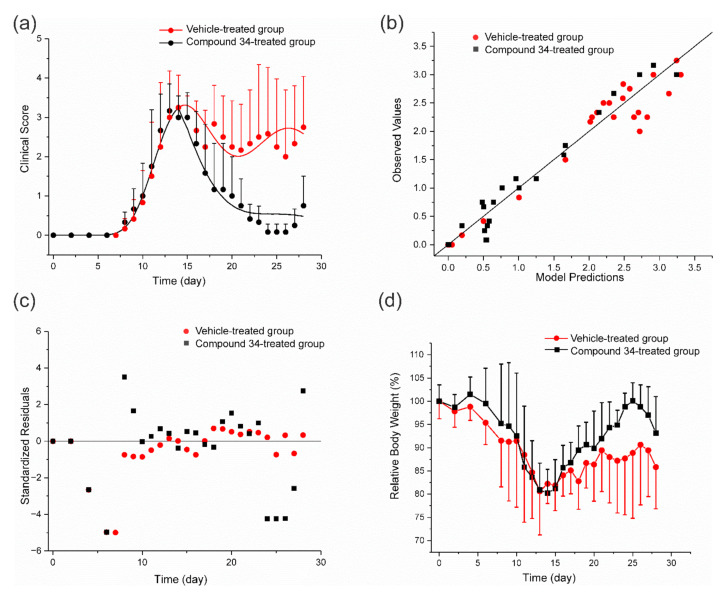
The results of the fitting of the PK/PD EAE progression model to the data and relative body weights of mice from the study and control groups; (**a**) time courses of the mean (+SD) *CS* in mice with EAE treated with compound **34** at an IP dose of 50 mg·kg^−1^ once a day for 15 days starting from the 14th day post-immunization. Curves depict model fittings and symbols are measured values; (**b**,**c**) are diagnostic plots of the PK/PD EAE progression model; (**d**) mean (±SD) body weight in mice with EAE (*n* = 6).

The results of the fitting of the PK/PD EAE progression model to the experimental data can be seen in Figure 6.

As it is shown in the Figure 6, the model reasonably captured the experimental data. It well described the initial rapid increase in *CS* observed after 7th day post-immunization, the subsequent temporary remission that occurred between 14th and 21st day, and a relapse of the disease after the 21st day of the experiment. The mean relative body weight was not significantly different between the experimental and control groups. In both groups, there was a drop observed in body weight after 7th day post-immunization, when the first symptoms of the disease occurred, and a subsequent gradual increase in body weight that seemed to be steeper in compound **34**-treated group. Estimates of EAE progression and PD parameters of compound **34** are presented in Table 4.

Compound **34** displayed a high inhibitory activity on disease progression with the *IC*_50_*CS*_ value of 0.007 mg·L^−1^ that was of the same order of magnitude as in the experiment involving the CIA progression model (Table 3). The diagnostic plots indicate reasonable fitting of the model to the data. Residuals were evenly distributed around zero; however, the model poorly captured the *CS* values measured after the 24th day, suggesting that not all significant aspects of EAE development might have been reflected in the structure of the proposed PK/PD disease progression model.

### 3.5. ConA-Induced Hepatitis Model

**Figure 7:**
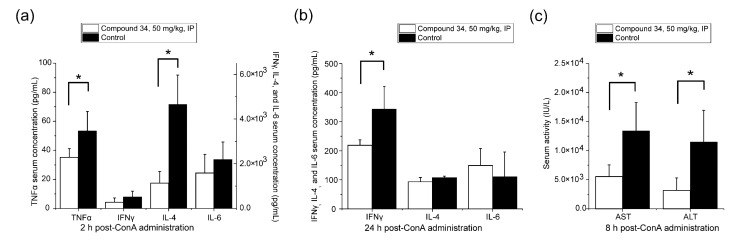
Mean (+SD) (**a**) serum TNFα, IFNγ, IL-4, and IL-6 concentrations at 2 h post-ConA administration (*n* = 4); (**b**) IFNγ, IL-4, and IL-6 concentrations at 24 h post-ConA administration (*n* = 4); (**c**) serum AST and ALT activities at 8 h post-ConA dosing (*n* = 4). Study group received compound **34** at an IP dose of 50 mg·kg^−1^ 30 min prior to the ConA dosing. * *p* < 0.05, a Student’s *t*-test.

As was expected, IV administration of ConA in BALB/c mice caused an acute immune-mediated hepatitis that was manifested by the increased concentrations of pro-inflammatory cytokines and other signaling molecules associated with inflammation that was accompanied by a rise in serum transaminase activities, namely AST and ALT, which are biomarkers of liver cell damage [31]. Figure 7 shows concentrations of inflammatory mediators and transaminase activities in serum of mice with ConA-induced AIH at different time points post-ConA administration in compound **34**-treated group and the vehicle-treated control group. 

At 2 h after ConA injection, in the compound **34**-treated group, the concentration of TNFα was 34% lower and for IL-4 it was 76% lower compared to the control group, while the levels of IL-6 and IFNγ were not significantly different at this time point compared to the control group. In contrast, at 24 h post-ConA dosing, the concentration of IFNγ in serum was 36% lower in the compound **34**-treated group compared to the control group and the levels of IL-4 and IL-6 were not different between the treatment and control group, whereas levels of TNFα were not measurable at this time point in both groups. AST and ALT serum activities measured at 8 h post-ConA injection were reduced by 59% and 73%, respectively, in the compound **34**-tretaed group compared to the control group. The results of this experiment indicate that compound **34** exhibits a strong hepatoprotective activity at an IP dose of 50 mg·kg^−1^ by decreasing the levels of inflammatory mediators that are engaged in the pathogenesis of ConA-induced AIH in mice. 

## 4. Discussion

In the present study, using a PK/PD disease progression modeling approach, we mathematically described and assessed the effects of a new non-selective PDE inhibitor, compound **34**, with a strong PDE1B, PDE3A, PDE4B, and PDE7A suppressing activity. To this end, we employed three animal models of ADs, namely CIA in rats [24], a MOG_35–55_-induced EAE in mice [10], and a ConA-induced hepatitis in mice [20]. ADs, such as RA or MS, represent a growing economic burden for societies around the world and have a profound negative impact on the health-related quality of life of people suffering from these diseases [32]. Current treatment strategies of ADs aim at induction of remission when the disease is not active, and its maintaining or minimization of the disease activity. For the induction of remission, the most frequently used medications are corticosteroids administered at high doses that are given at the disease onset or in the case of flare-up. These medications are often maintained at lower doses in order to sustain the remission [33]. Immunosuppressive drugs are the second large group of compounds used to maintain the remission that, in addition, allow for the reduction of glucocorticoids’ doses. Both corticosteroids and immunosuppressives are burdened with severe side effects, including but not limited to metabolic, hematologic, and immune-related adverse reactions [34]. The rapidly growing group of medicines used for the treatment of ADs are biological drugs, such as antibodies and receptors targeting cytokines and immune cells; however, also these agents are not always effective and may cause severe adverse effects [35]. Therefore, there is an urgent need for new effective and safe therapies of ADs. 

PDE inhibitors, especially those inhibiting PDE1, PDE3, PDE4, and PDE7, are a group of compounds that has been extensively tested in recent years as possible treatment option of ADs and other inflammatory diseases of different etiology [5,36,37]. Both selective and dual PDE4 and PDE7 inhibitors were previously shown to be effective in MOG_35–55_-induced EAE [8,11], CIA [17,18], and ConA-induced hepatitis [38,39]. Recently, we demonstrated that simultaneous inhibition of PDE3, PDE4, and PDE7 may constitute a possible therapeutic strategy of AIH [40,41]. This study is a continuation of our previous investigations aiming at the assessment and description of pharmacological effects of compounds able to inhibit relevant PDEs in animal models of immune-mediated disorders using mathematical modeling approaches. 

In this work, we assessed a new purine-2,6-dione derivative that was designed as a strong PDE4 and PDE7 inhibitor and preliminarily assessed in our previous study [25]. It was selected for the further in vitro and in vivo assessment based on its high inhibitory activity against PDE4 and PDE7 tested in vitro, and a profound TNFα-inhibitory potency assessed in a rat model of LPS-induced endotoxemia [25]. This compound, given at a single IP dose of 50 mg·kg^−1^ in rats, reduced maximum plasma TNFα concentrations by 84% at 1.5 h post-LPS dosing. It has to be noted that compound **34** has a similar chemical structure to that previously investigated by our group PDE inhibitor, GRMS-55. Compound **34**, in contrast to GRMS-55, has two additional hydroxyl substituents in the phenyl ring. Both compounds, based on their pharmacological activity, were selected from the same library of newly designed theophylline derivatives that was preliminarily assessed in our previous studies [25]. GRMS-55 was effective in several animal models of immune-related disorders, i.e., CIA in rats, LPS-induced endotoxemia in rats, and ConA-induced hepatitis in mice [28,41]. 

In this study, compound **34** was evaluated in vitro as an inhibitor of PDE1 and PDE3, since these isoenzymes, beside PDE4 and PDE7, are located in a variety of immune cells and are involved in regulatory mechanisms in these cells [42,43]. Despite a high similarity in the structure, compound **34** turned out to be about two times more potent as a PDE7A inhibitor and 19 times more active as a PDE4B inhibitor compared to GRMS-55 [28]. Compound **34** displayed a high inhibitory activity against PDE1B and also high, but lower than a strong selective PDE3-inhibitor milrinone, potency against PDE3A. Therefore, the observed efficacy of compound **34** in vivo may be possibly attributed to the high PDE-inhibitory activity of this compound, since simultaneous inhibition of these immune-related PDE isoforms was previously shown to be associated with amelioration of the symptoms in in vivo models of immune-related disorders [28,41].

Subsequently, compound **34** was assessed in the CIA in rats. The first symptoms of arthritis in rats, such as redness and swelling of joints, developed at the 12th day post the first immunization with porcine collagen type II. These symptoms developed until the 20th day of the experiment. The time of disease onset and the time of maximum paw edema in rats were similar to the results of previous studies involving this animal model of RA [28,44]. In the present study, compound **34** displayed a high activity in inhibiting the progression of CIA, with the *IC*_50_*Paw*_ value of 0.001 mg·L^−1^ that was estimated using the PK/PD arthritis progression model. This value is around 260 times lower than that for GRMS-55, which was estimated in our previous study using the same PK/PD model which indicates much higher potency of compound **34** compared to GRMS-55 [28]. Moreover, the *IC*_50_*Paw*_ value estimated using the PK/PD model for compound **34** in suppressing paw edema was 10 times lower than that for rolipram, a strong PDE4-selective inhibitor [28]. In the light of these findings and based on the PDE-inhibitory profile of the investigated compounds, it is highly likely that a strong simultaneous inhibition of PDE1, PDE3, PDE4, and PDE7 potentiates the efficacy of the studied PDE inhibitor, and this treatment strategy may be beneficial compared to selective inhibition of relevant PDE isoenzymes. Indeed, some previous studies showed that dual or non-selective PDE inhibitors may display stronger effects in ameliorating symptoms of ADs than selective PDE4 or PDE7 inhibitors [16,17,45]. PDE1, PDE3, PDE4, and PDE7 are isoforms of PDE present in many immune cells, including but not limited to T cells, monocytes, and macrophages that are engaged in the pathogenesis of ADs [42,43]. The possible explanation of this phenomenon may be that simultaneous inhibition of these isoforms causes a high rise in the intracellular cAMP concentrations in immune cells due to the suppression of cAMP hydrolysis in these cells leading to a subsequent phosphorylation of protein kinase A that participate in the regulation of expression of genes coding inflammatory mediators, such as cytokines [46,47]. In our previous investigation, we found out that the simultaneous inhibition of PDE3, PDE4, and PDE7 that are present in T cells may constitute an alternative treatment strategy of T cell-mediated ADs, such as AIH [40,41]. 

Disease progression modeling is an expanding field related to mathematical modeling that aids in understanding the determinants of disease development and drug actions. This approach is useful in the assessment of drug effects and in translating the results of preclinical studies into the clinic. In this study, we developed a new PK/PD EAE progression model of a PDE inhibitor using the *CS* as a surrogate marker of disease progression in mice. A similar modeling approach was previously used in disease progression models of schizophrenia by using the positive and negative syndrome scale [48] and in arthritis progression modeling using the disease activity score as a marker of disease progression [49]. In this paper, we propose a new PK/PD model structure to describe EAE progression in mice. As far as we know, this is the first attempt of a mathematical description of EAE dynamics and progression. EAE is the animal model most commonly used to study the efficacy of potential drug candidates for the treatment of MS. It is characterized by paralysis, inflammation, and demyelination of the CNS. Its development is mediated by myelin-specific CD4+ T cells [9,10,30]. The proposed mathematical model is composed of a simple PK model for compound **34** linked to a biophase model, which is subsequently combined with an indirect response model type I [29], in which the zero-order rate constant representing the disease progression is simultaneously stimulated by the signal from two transit compartments and inhibited by the concentration of the drug in the biophase. This structure of the PK/PD disease progression model is consistent with the mechanism of action of PDE inhibitors that, upon distribution to immune cells (e.g., T cells), inhibit production of pro-inflammatory cytokines responsible for EAE progression [8,50]. In addition, the model structure reflects the multistage process leading to EAE development that is initiated at the time of immunization with MOG_35–55_ peptide in combination with a complete Freund′s adjuvant. Upon immunization, an expansion and differentiation of MOG-specific autoimmune T cells is observed [10], which is captured in the proposed PK/PD model by signal transduction involving 38 transit steps. Stimulation of the *k_dis_* constant by the sum of signals from two different transit compartments (20th and 38th) accounts for the initial rise in *CS* observed around 8th day of the experiment and a subsequent temporary remission observed after 14th day post-immunization [30]. This disease progression model, upon some modifications, may be helpful in the assessment of drugs with different mechanism of action than PDE inhibition and also in translating the results of preclinical investigations into the clinic, e.g., guiding the first-in-human dose selection for clinical trials or optimizing the dosing schedules of existing medications. The major limitations of this model, as well as the PK/PD CIA progression model, are that they do not take into consideration a possible protein binding of the drug in serum, compound **34** was administered only at one dose level, and pharmacokinetics of compound **34** was assessed in healthy mice and rats after single administration. Therefore, the models do not take into account possible changes in pharmacokinetics of the compound that might have occurred due to the disease development and multiple dosing. In this investigation, female rodents were used, since female mice and rats display a more exaggerated immune response after immunization compared to male rodents and produce higher levels of antibodies and activated T lymphocytes, resulting in a much higher incidence of autoimmune disorders [51]. The lack of heathy (non-immunized) animals in the pharmacodynamic experiments is also a limitation of this study, since measurements of body weight and paw size in these animals could provide information regarding natural paw growth rate and natural increase in body weight.

Compound **34** exhibited a high potency in attenuating symptoms of EAE in mice compared to the vehicle-treated control group; however, in the study group a relapse of disease was observed after the 26th day of the experiment, suggesting a loss of efficacy of the drug with time. This model does not account for this observation and, because of that, some discrepancies are evident between observed and predicted values of *CS* on the later days of the experiment. The possible reasons for the observed loss of efficacy may be due to feedback mechanisms, such as up-regulation of PDEs or autoinduction of metabolism of compound **34** upon multiple administration, leading to decreased exposure to the drug. However, it should be noted that a similar phenomenon was observed in the study involving PDE7 inhibitor administered once daily in the EAE model in mice [15]. In that study, the therapeutic effect of the investigated PDE7 inhibitor was lost after 31st day post-immunization, i.e., on the 16th day of the drug administration. This observation suggests that the reduction of efficacy with time may be related to prolonged PDE7 inhibition. In another study, rolipram alone and in combination with BRL—50481, a selective PDE7 inhibitor, and a novel quinazoline derivative with PDE7 inhibitory activity, TC3.6, diminished T-cell proliferation, reduced expression of pro-inflammatory mediators, and reduced cellular infiltrates in the CNS. These effects were accompanied by the amelioration of the clinical symptoms of EAE in mice [8].

In ConA-induced hepatitis in mice, compound **34** exhibited similar effects to its analog GRMS-55, which was assessed in our previous investigations [28,41]. It reduced ALT and AST activities in serum and diminished concentrations of TNFα, IFNγ, and IL-4. These results suggest that the observed anti-inflammatory effect of compound **34** in immune-related disorders is possibly due to its PDE-inhibitory activity leading to the regulation of expression of inflammatory mediators. Previously, selective inhibition of PDE3 [52], PDE4 [39], and PDE7 [38] was shown to diminish the outcomes of ConA-induced hepatitis in mice. Using selective inhibitors of these enzymes, we previously confirmed this observation and, using a newly designed PK/PD model, assessed a partial contribution of these isoforms of PDE to the overall hydrolytic activity against cAMP in immune cells that are engaged in the development of ConA-induced hepatitis [40]. 

According to the result of the pharmacokinetic study, the half-life of compound **34** is relatively short in both mice and rats, and equals 13.6 min and 14.7 min in these species. It is considerably shorter compared to the half-life of its analog, GRMS-55, which was around 30 min in both rats and mice. It may be concluded that two additional hydroxyl substituents in the phenyl ring resulted in the reduction of metabolic stability of the compound. Thus, further efforts in designing new PDE inhibitors in the group of theophylline derivatives should aim at increasing their metabolic stability while maintaining the pharmacological activity and low toxicity.

## 5. Conclusions

In this study, a new PK/PD EAE progression model of a PDE inhibitor was developed that reasonably captured *CS*-time courses in mice with EAE treated with a new investigational PDE inhibitor, compound **34**, or vehicle alone. In addition, a previously developed PK/PD arthritis progression model was used to assess the effects of this compound in CIA in rats. The compound displayed a strong activity in ameliorating symptoms of CIA in rats with the *IC*_50_*Paw*_ of 0.001 mg·L^−1^ and in EAE in mice with the *IC*_50_*CS*_ of 0.007 mg·L^−1^. Additionally, compound **34** strongly reduced serum concentrations of TNFα, IFNγ, and IL-4, and diminished serum ALT and AST activities in ConA-induced hepatitis in mice. Interestingly, these beneficial effects occurred despite a relatively short half-life of compound **34** in both mice and rats. The newly designed PK/PD EAE progression model, as well as the PK/PD arthritis progression model of compound **34**, upon modifications, may be used in future studies to assess new investigational compounds or in translating the results of preclinical investigations into the clinic.

## Figures and Tables

**Table 1:** Inhibitory activity of compound **34** and respective reference compounds against PDE1B, 3A, 4B, and 7A.

Compound	IC_50_ (µM) Against PDE Isoform (CV%)			
	PDE1B	PDE3A	PDE4B	PDE7A
Compound **34**	0.21 (29.2)	0.78 (8.3)	1.4 ^c^	3.2 ^c^
GRMS-55	2.5 ^b^	13.4 ^b^	26.9 ^b^	7.3 ^b^
Reference compound ^a^	7.5 ^b^	0.2 ^b^	1.1 ^b^	2.1 ^b^

^a^ Vinpocetine for PDE1B, milrinone for PDE3A, rolipram for PDE4B, and BRL—50481 for PDE7A. ^b^ data from [28]; ^c^ data from [25].

**Table 2:** PK parameters of compound **34** in rats and mice.

Parameter	Brief Description	Species	
		Rat	Mouse
		Final Estimate (CV%)	Final Estimate (CV%)
*Vd/F* (L·kg^−1^)	Apparent volume of distribution	8.70 (18.0)	21.69 (34.6)
*k_a_* (min^−1^)	Absorption rate constant	0.58 (159.2)	0.15 (65.6)
*AUC* (mg·L^−1^·min)	Area under the concentration-time curve	45.1 (12.6)	49.1 (12.3)
*t*_0.5_ (min)	Elimination half-life	13.6 (13.5)	14.7 (31.2)
*C_max_* (mg·L^−1^)	Maximum serum concentration	1.82 (28.9)	1.37 (14.2)
*CL_T_/F* (L·min^−1^·kg^−1^)	Apparent clearance	0.44 (12.6)	1.02 (12.3)

Compound **34** exhibited a rapid elimination in both rats and mice with the half-life of 13.6 min in rats and 14.7 min in mice.

**Table 3:** Arthritis progression and PD parameters of compound **34** in rats.

Parameter	Brief Description	Final Estimate (CV%)
*k_out_* (day^−1^)	Degradation of precursor rate constant	0.207(37.9)
*k_deg_* (day^−1^)	Loss of production rate constant	0.035 (18.4)
*t_onset_* (day)	Time of disease onset	8.70 (9.5)
*τ* (day)	Mean transit time	0.807 (34.5)
*IC*_50_*Paw*_ (mg·L^−1^)	Concentration producing 50% of maximum inhibition	0.001 (354.5)
*k_grow_* (day^−1^)	Natural growth rate constant	0.212 (37.8)
*k*_*in(*0*)C*_ (day^−1^)	Initial value of precursor production variable in the compound **34**-treated group	0.748 (28.2)
*k*_*in(*0*)S*_ (day^−1^)	Initial value of precursor production variable in vehicle-treated group	0.752 (28.9)

**Table 4:** EAE progression and PD parameters of compound **34** in mice.

**Parameter**	**Brief Description**	**Estimate (CV%)**
*τ_P_* (day)	Mean transit time of disease progression precursor	0.6564 (1.6)
*k_dis_* (day^−1^)	Disease progression rate constant	16.46 (15.1)
*k_rem_* (day^−1^)	Disease remission rate constant	0.3654 (17.6)
*k_eo_* (day^−1^)	Serum–effect-site equilibration rate constant	0.3287 (95.2)
*P*_1_(0)	Initial value of the first disease progression precursor	1 (fix)
*IC*_50_*CS*_ (mg·L^−1^)	Compound **34** concentration resulting in 50% inhibition of disease progression	0.0069 (17.0)

## Data Availability

The data presented in this paper are available upon reasonable request from the corresponding author. The PK/PD EAE progression model code of compound **34** is available in Supplementary Materials.

## Supplementary Materials

The ADAPT5 model code for the PK/PD EAE progression model of compound **34** can be downloaded at: https://www.mdpi.com/article/10.3390/pharmaceutics14051090/s1.
