# Supplementary material for: Pharmacokinetic/Pharmacodynamic Evaluation of a New Purine-2,6-Dione Derivative in Rodents with Experimental Autoimmune Diseases
Source: Pharmaceutics. 2022 May 19;14(5):1090. doi: 10.3390/pharmaceutics14051090 (PMC9147171; doi:10.3390/pharmaceutics14051090)
Supplement: Supplementary file 1 [file pharmaceutics-14-01090-s001.zip › pharmaceutics-1713189-supplementary.pdf]

```
C*********************************************************************
C                           ADAPT                                    *
C                         Version 5                                  *
C*********************************************************************
C                                                                    *
C     The PK/PD EAE progression model of compound 34
C     Developed by Dr. Artur Świerczek
C                                                                    *
C*********************************************************************

C####################################################################C

        Subroutine SYMBOL
        Implicit None

        Include 'globals.inc'
        Include 'model.inc'

CC
C--------------------------------------------------------------------C
C                   Enter as Indicated                               C
C--------------------------------------------------------------------C

      NDEqs   =  44   ! Enter # of Diff. Eqs.
      NSParam =  5   ! Enter # of System Parameters.
      NVparam =  2   ! Enter # of Variance Model Parameters.
      NSecPar =  0   ! Enter # of Secondary Parameters.
      NSecOut =  0  ! Enter # of Secondary Outputs (not used).
      Ieqsol  =  1  ! Indicates a built-in compartment model.
      Descr = 'PK/PD encephalomyelitis progression model of compound 34'

CC
C--------------------------------------------------------------------C
C     Enter Symbol for Each System Parameter (eg. Psym(1)='Kel')       C
C----c---------------------------------------------------------------C


          PSym(1) = 'tau'
          PSym(2) = 'kin'
          PSym(3) = 'kout'
          PSym(4) = 'ic50'
          PSym(5) = 'kt'


CC
C--------------------------------------------------------------------C
C    Enter Symbol for Each Variance Parameter {eg: PVsym(1)='Sigma'}   C
C----c---------------------------------------------------------------C

       PVsym(1) = 'SDinter'
       PVsym(2) = 'SDslope'
CC
C--------------------------------------------------------------------C
C    Enter Symbol for Each Secondary Parameter {eg: PSsym(1)='CLt'}    C
C----c---------------------------------------------------------------C
```

```
C------------------------------------------------------------------------C
C------------------------------------------------------------------------C
C
        Return
        End

C########################################################################C

        Subroutine DIFFEQ(T,X,XP)
        Implicit None

        Include 'globals.inc'
        Include 'model.inc'

      Real*8 T,X(MaxNDE),XP(MaxNDE)
      Real*8 Tau, kin, kout, Response, P1, P2, P3, P4, P5, P6, P7, P8
      Real*8 P9, P10, P11, P12, P13, P14, P15, P16, P17, P18, P19, P20
      Real*8 P21, P22, P23, P24, P25, P26, P27, P28, P29, P30
      Real*8 P31, P32, P33, P34, P35, P36, P37, P38
      Real*8 dResponse, dP1, dP2, dP3, dP4, dP5, dP6, dP7, dP8, dP9,dP20
      Real*8 dP10, dP11, dP12, dP13, dP14, dP15, dP16, dP17, dP18, dP19
      Real*8 dP21, dP22, dP23, dP24, dP25, dP26, dP27, dP28, dP29, dP30
      Real*8 dP31, dP32, dP33, dP34, dP35, dP36, dP37, dP38
      Real*8 dAp, dXpl, Cp, Xpl, Ap, IC50, Response34, dResponse34
      Real*8 Dt1, Dt2, dT3, T1, kt


C------------------------------------------------------------------------C
C     Enter Differential Equations Below  {e.g.  XP(1) = -P(1)*X(1) }  C
C----c-------------------------------------------------------------------C

         Tau = P(1) !Mean transit time of disease progression precursor
         kin = P(2) !Disease progression rate constant
         kout = P(3) !Disease remission rate constant
         IC50 = P(4) !Compound 34 concentration resulting in 50% inhibition of disease 
progression
         kt = P(5) !Serum-effect-site equilibration rate constant

      P1 = X(1)
      P2 = X(2)
      P3 = X(3)
      P4 = X(4)
      P5 = X(5)
      P6 = X(6)
      P7 = X(7)
      P8 = X(8)
      P9 = X(9)
      P10 = X(10)
      P11 = X(11)
      P12 = X(12)
      P13 = X(13)
      P14 = X(14)
      P15 = X(15)
      P16 = X(16)
      P17 = X(17)
      P18 = X(18)
      P19 = X(19)
      P20 = X(20)
```

```
      P21 = X(21)
      P22 = X(22)
      P23 = X(23)
      P24 = X(24)
      P25 = X(25)
      P26 = X(26)
      P27 = X(27)
      P28 = X(28)
      P29 = X(29)
      P30 = X(30)
      P31 = X(31)
      P32 = X(32)
      P33 = X(33)
      P34 = X(34)
      P35 = X(35)
      P36 = X(36)
      P37 = X(37)
      P38 = X(38)

      Response = X(39)
      Response34 = X(40)

      Ap = X(41)
      Xpl = X(42)
      T1 = X(43)
!  Differential equations
          ! Pharmacokinetics:
      dAp=-216*Ap
      dXpl=216*Ap-67.7*Xpl
          Cp=Xpl/21.69
      dT1=(Cp-T1)*kt
          ! Pharmacodynamics and disease progression:
      dResponse = kin*(P20+P38)-kout*Response
      dResponse34 = kin*(P20+P38)*(1-T1/(IC50+T1))-kout*Response34

      dP1 = (-1/Tau*P1)
      dP2 = (P1-P2)/Tau
      dP3 = (P2-P3)/Tau
      dP4 = (P3-P4)/Tau
      dP5 = (P4-P5)/Tau
      dP6 = (P5-P6)/Tau
      dP7 = (P6-P7)/Tau
      dP8 = (P7-P8)/Tau
      dP9 = (P8-P9)/Tau
      dP10 = (P9-P10)/Tau
      dP11 = (P10-P11)/Tau
      dP12 = (P11-P12)/Tau
      dP13 = (P12-P13)/Tau
      dP14 = (P13-P14)/Tau
      dP15 = (P14-P15)/Tau
      dP16 = (P15-P16)/Tau
      dP17 = (P16-P17)/Tau
      dP18 = (P17-P18)/Tau
      dP19 = (P18-P19)/Tau
      dP20 = (P19-P20)/Tau
      dP21 = (P20-P21)/Tau
      dP22 = (P21-P22)/Tau
      dP23 = (P22-P23)/Tau
```

```
dP24 = (P23-P24)/Tau
dP25 = (P24-P25)/Tau
dP26 = (P25-P26)/Tau
dP27 = (P26-P27)/Tau
dP28 = (P27-P28)/Tau
dP29 = (P28-P29)/Tau
dP30 = (P29-P30)/Tau
dP31 = (P30-P31)/Tau
dP32 = (P31-P32)/Tau
dP33 = (P32-P33)/Tau
dP34 = (P33-P34)/Tau
dP35 = (P34-P35)/Tau
dP36 = (P35-P36)/Tau
dP37 = (P36-P37)/Tau
dP38 = (P37-P38)/Tau

XP(1)= dP1
XP(2)= dP2
XP(3)= dP3
XP(4)= dP4
XP(5)= dP5
XP(6)= dP6
XP(7)= dP7
XP(8)= dP8
XP(9)= dP9
XP(10)= dP10
XP(11)= dP11
XP(12)= dP12
XP(13)= dP13
XP(14)= dP14
XP(15)= dP15
XP(16)= dP16
XP(17)= dP17
XP(18)= dP18
XP(19)= dP19
XP(20)= dP20
XP(21)= dP21
XP(22) = dP22
XP(23) = dP23
XP(24) = dP24
XP(25) = dP25
XP(26) = dP26
XP(27) = dP27
XP(28) = dP28
XP(29) = dP29
XP(30) = dP30
XP(31) = dP31
XP(32) = dP32
XP(33) = dP33
XP(34) = dP34
XP(35) = dP35
XP(36) = dP36
XP(37) = dP37
XP(38) = dP38
XP(39) = dResponse !Control group
XP(40) = dResponse34 !Compound 34-treated group
XP(41) = dAp
XP(42) = dXpl
```

```
      XP(43) = dT1


C----------------------------------------------------------------------C
C----------------------------------------------------------------------C
C
        Return
        End

C######################################################################C

   Subroutine OUTPUT(Y,T,X)
        Implicit None

        Include 'globals.inc'
        Include 'model.inc'

        Real*8 Y(MaxNOE),T,X(MaxNDE)
        Real*8
CC
C----------------------------------------------------------------------C
C     Enter Output Equations Below   {e.g.  Y(1) = X(1)/P(2) }         C
C----c-----------------------------------------------------------------C

C  Note: X(1), X(2) and X(3) are the amounts in the central, absorption
C     and peripheral compartments, respectively.

       Y(1)=X(39)
       Y(2)=X(40)

C----------------------------------------------------------------------C
C----------------------------------------------------------------------C
C
        Return
        End

C######################################################################C

        Subroutine VARMOD(V,T,X,Y)
        Implicit None

        Include 'globals.inc'
        Include 'model.inc'

        Real*8 V(MaxNOE),T,X(MaxNDE),Y(MaxNOE)

CC
C----------------------------------------------------------------------C
C       Enter Variance Model Equations Below                           C
C        {e.g. V(1) = (PV(1) + PV(2)*Y(1))**2 }                        C
C----c-----------------------------------------------------------------C

        V(1) = (PV(1) + PV(2)*Y(1))**2
        V(2) = (PV(1) + PV(2)*Y(2))**2


C----------------------------------------------------------------------C
C----------------------------------------------------------------------C
```

```fortran
        Return
        End

C######################################################################C

        Subroutine COVMOD(Pmean, ICmean, PC)
C  Defines any covariate model equations (MLEM, ITS)
        Implicit None

        Include 'globals.inc'
        Include 'model.inc'

        Real*8 PC(MaxNCP)
        Real*8 Pmean(MaxNSP+MaxNDE), ICmean(MaxNDE)

CC
C----------------------------------------------------------------------C
C     Enter # of Covariate Parameters                                  C
C----c-----------------------------------------------------------------C

        NCparam = 0    ! Enter # of Covariate Parameters.

CC
C----------------------------------------------------------------------C
C   Enter Symbol for Covariate Params {eg: PCsym(1)='CLRenal'}         C
C----c-----------------------------------------------------------------C


CC
C----------------------------------------------------------------------C
C   For the Model Params. that Depend on Covariates Enter the Equation C
C         {e.g. Pmean(1) =  PC(1)*R(2) }                               C
C----c-----------------------------------------------------------------C


C----------------------------------------------------------------------C
C----------------------------------------------------------------------C
C
        Return
        End

C######################################################################C

Subroutine POPINIT(PmeanI,ICmeanI,PcovI,ICcovI, PCI)
C  Initial parameter values for population program parameters (ITS, MLEM)

        Implicit None

        Include 'globals.inc'
        Include 'model.inc'

        Integer I,J
        Real*8 PmeanI(MaxNSP+MaxNDE), ICmeanI(MaxNDE)
        Real*8 PcovI(MaxNSP+MaxNDE,MaxNSP+MaxNDE), ICcovI(MaxNDE,MaxNDE)
        Real*8 PCI(MaxNCP)

CC
C----------------------------------------------------------------------C
```

```
C  Enter Initial Values for Population Means                        C
C          {  e.g. PmeanI(1) = 10.0    }                            C
C----c--------------------------------------------------------------C


CC
C-------------------------------------------------------------------C
C   Enter Initial Values for Pop. Covariance Matrix (Lower Triang.)    C
C         {  e.g. PcovI(2,1) = 0.25    }                            C
C----c--------------------------------------------------------------C


CC
C-------------------------------------------------------------------C
C   Enter Values for Covariate Model Parameters                     C
C         {  e.g. PCI(1) = 2.0    }                                 C
C----c--------------------------------------------------------------C


C-------------------------------------------------------------------C
C-------------------------------------------------------------------C
C
        Return
        End

C###################################################################C

        Subroutine PRIOR(Pmean,Pcov,ICmean,ICcov)
C  Parameter mean and covariance values for MAP estimation (ID,NPD,STS)
        Implicit None

        Include 'globals.inc'
        Include 'model.inc'

        Integer I,J
        Real*8 Pmean(MaxNSP+MaxNDE), ICmean(MaxNDE)
        Real*8 Pcov(MaxNSP+MaxNDE,MaxNSP+MaxNDE), ICcov(MaxNDE,MaxNDE)

CC
C-------------------------------------------------------------------C
C  Enter Nonzero Elements of Prior Mean Vector                      C
C          {  e.g. Pmean(1) = 10.0    }                             C
C----c--------------------------------------------------------------C


CC
C-------------------------------------------------------------------C
C   Enter Nonzero Elements of Covariance Matrix (Lower Triang.)       C
C         {  e.g. Pcov(2,1) = 0.25    }                             C
C----c--------------------------------------------------------------C


C-------------------------------------------------------------------C
C-------------------------------------------------------------------C
C
        Return
        End
```

```
C#######################################################################C

        Subroutine SPARAM(PS,P,IC)
        Implicit None

        Include 'globals.inc'

        Real*8 PS(MaxNSECP), P(MaxNSP+MaxNDE), IC(MaxNDE)

CC
C-----------------------------------------------------------------------C
C       Enter Equations Defining Secondary Paramters                    C
C           {  e.g.  PS(1) = P(1)*P(2)   }                              C
C----c------------------------------------------------------------------C


C-----------------------------------------------------------------------C
C-----------------------------------------------------------------------C
C
        Return
        End

C#######################################################################C

        Subroutine AMAT(A)
        Implicit None

        Include 'globals.inc'
        Include 'model.inc'

        Integer I,J
        Real*8 A(MaxNDE,MaxNDE)

        DO I=1,Ndeqs
           Do J=1,Ndeqs
              A(I,J)=0.0D0
           End Do
        End Do

CC
C-----------------------------------------------------------------------C
C    Enter non zero elements of state matrix  {e.g.  A(1,1) = -P(1) }  C
C----c------------------------------------------------------------------C



C-----------------------------------------------------------------------C
C-----------------------------------------------------------------------C
C
        Return
        End

C#######################################################################C
```
